# Die Beteiligung von Betroffenen und Angehörigen am Deutschen Zentrum für Psychische Gesundheit

**DOI:** 10.1007/s00115-021-01249-z

**Published:** 2022-01-05

**Authors:** Isabel Dziobek, Myriam Bea, Benjamin Drechsel, Rüdiger Hannig, Andreas Heinz, Silke Lipinski, Isabell Schick

**Affiliations:** 1 Klinische Psychologie Sozialer Interaktion, Institut für Psychologie, Humboldt-Universität zu Berlin, Unter den Linden 6, 10099 Berlin, Deutschland; 2 ADHS Deutschland e. V., Berlin, Deutschland; 3 Universitätsklinikum Heidelberg, Heidelberg, Deutschland; 4 Bundesverband der Angehörigen psychisch erkrankter Menschen e. V., Bonn, Deutschland; 5 Klinik für Psychiatrie und Psychotherapie CCM, Charité – Universitätsmedizin Berlin, Berlin, Deutschland; 6 Aspies e.V. – Menschen im Autismusspektrum, Berlin, Deutschland; 7 Rettungs-Ring e. V., Neu-Ulm, Deutschland

Patient and Public Involvement (PPI) zielt darauf ab, Betroffene und Angehörige als Mitgestalter in den Forschungsprozess zu integrieren. Dafür gibt es nicht nur ethische und rechtliche Grundlagen [1], sondern PPI kann Forschung qualitativ besser, relevanter, glaubwürdiger machen und sogar Kosten reduzieren [2]. Konsequenterweise fordern zunehmend Forschungsförderer und wissenschaftliche Journale die Beteiligung von Betroffenen und Angehörigen in allen Phasen des Forschungsprozesses. Trotzdem wird PPI bisher in der psychologisch-psychiatrischen Mainstreamforschung in Deutschland kaum realisiert [3, 4].

Das sich derzeit konstituierende Zentrum für Psychische Gesundheit (DZPG) will in bisher nicht dagewesenem Umfang die Beteiligung von Betroffenen und Angehörigen realisieren. Derzeit werden im Rahmen der 6‑monatigen Konzeptentwicklungsphase für das Zentrum die Weichen dafür gestellt. Die historische Chance, die sich hierbei auftut, Forschung und Versorgung im Bereich psychische Gesundheit in Deutschland zu demokratisieren und weiter zu verbessern, wird zukunftsweisend ergriffen. Wissenschaftliche Arbeiten sollen stärker als bisher die Bedürfnisse der Betroffenen und Angehörigen berücksichtigen und von der Antragstellung bis zur Kommunikation von DZPG-Studienergebnissen sollen stets das aus Erfahrung entstandene Wissen und die Perspektiven von Betroffenen und Angehörigen einfließen.

Noch vor dem Start der Konzeptentwicklungsphase wurde im Sommer 2021 ein trialogischer Zentrumsrat etabliert, um Betroffene und Angehörige bereits auch bei der Erarbeitung von Leitungsstruktur und inhaltlicher Ausrichtung des DZPG einzubinden. Der Zentrumsrat setzt sich aus insgesamt 18 Betroffenen und Angehörigen – Vertreter von Selbsthilfe‑/Angehörigenverbänden und Privatpersonen – sowie Forschern der zukünftigen DZPG-Standorte zusammen. Durch je zwei Betroffenen‑/Angehörigenvertreter des trialogischen Zentrumsrats werden Anliegen und eigene erarbeitete Inhalte, gleich wie von den Standorten, in den Lenkungsausschuss eingebracht. In diesem haben Betroffene und Angehörige insgesamt 25 % des Stimmanteils bei allen Entscheidungen, sodass sie wirklich Gehör finden und ihre Stimme Gewicht hat.

Zudem fließen bereits jetzt Positionen und Anliegen der Betroffenen und Angehörigen in die Inhalte der Forschungslinien, die für das DZPG derzeit definiert werden. Der trialogische Zentrumsrat wirkt in sämtlichen Arbeitsgruppen mit und setzt sich dafür ein, dass Themen wie Peer-Support, Entstigmatisierung, Salutogenese, Umgang von Angehörigen mit Suiziden und partizipatives Forschen in der klinischen Psychologie und Psychiatrie einen festen Platz in der Forschungsagenda der kommenden Jahre in Deutschland haben.

Um die von Betroffenen und Angehörigen gesehenen Forschungsnotwendigkeiten in Deutschland zukünftig auf einer noch breiteren Basis berücksichtigen zu können, wünscht sich der Zentrumsrat die Realisation eines eigenständigen Forschungsprojekts, bei dem deutschlandweit Tausende Betroffene und Angehörige im Rahmen von Fokusgruppen und Online-Befragungen ihre Bedarfe an aufzugreifenden oder zu verstärkenden Forschungsthemen einbringen könnten.

Um neben inhaltlicher und struktureller Mitsprache PPI auch nachhaltig in der konkreten Forschung am DZPG zu implementieren, wird derzeit eine *Abteilung für Patient and Public Involvement* als unterstützende Infrastruktur entworfen. Vor allem durch die Einrichtung von Referentenstellen für die Koordination soll qualitativ hochwertige und kontinuierliche Unterstützung von Betroffenen und Angehörigen sowie Forschern für die partizipative Forschung des DZPG gewährleistet werden. Die Referenten sollen zentrumsübergreifend wie standortspezifisch sowohl den Aufbau partizipativer Komponenten als auch bei der Durchführung konkreter Projekte in allen Forschungslinien beraten und schulen. Des Weiteren sollen sie die Kommunikation zwischen den Erfahrungsexperten und Forschern herstellen und unterstützen, PPI-Werkzeuge bereitstellen und das Qualitätsmanagement für PPI am DZPG realisieren.

Durch weitere geplante PPI-Elemente sollen Erfahrungskompetenz und Perspektive von Betroffenen und Angehörigen in alle DZPG-finanzierten Projekte eingehen. So sollen Betroffene und Angehörige Mitsprache bei Ausschreibungen und Begutachtungskriterien haben und sie sollen als Gutachter in Auswahlverfahren von Forschungsanträgen eingesetzt werden. Daneben soll ein Förderformat für betroffenen-/angehörigeninitiierte Projekte eingerichtet werden.

Weiterhin wird angestrebt, dass bei Neubesetzungen von wissenschaftlichen Mitarbeiterstellen Erfahrungsexpertise der Bewerber als Betroffener oder Angehöriger im entsprechenden Forschungsfeld als positives Auswahlkriterium berücksichtigt wird.

Außerdem soll die Integration von PPI als eigener Forschungsschwerpunkt ins DZPG realisiert werden, z. B. könnte in Form einer Professur dem Bereich PPI am DZPG zu internationaler Sichtbarkeit und Vernetzung verholfen werden. Ein solcher Forschungsschwerpunkt würde PPI in der deutschen klinischen Psychologie und Psychiatrie zeitgemäßes Gewicht geben und kontinuierliche Neuentwicklungen garantieren.

In Deutschland gibt es im Bereich der psychischen Gesundheit im Vergleich zu Ländern mit starker PPI-Tradition wie England oder Australien derzeit kaum etablierte Strukturen für die Beteiligung von Erfahrungsexperten. Die für das DZPG geplanten PPI-Elemente sehen eine breit angelegte Infrastruktur und die Integration von Betroffenen und Angehörigen mit echter Entscheidungskompetenz in allen Gremien vor und bezeugen insofern, dass alle am DZPG beteiligten Standorte und Forscher progressiv auf die Chancen von PPI zugehen und es mit der Realisierung von PPI im DZPG ernst meinen; das DZPG setzt dazu an, nicht nur den internationalen Rückstand bezüglich PPI aufzuholen, sondern das Feld mit anzuführen.
